# Inhibition of the PI3K but not the MEK/ERK pathway sensitizes human glioma cells to alkylating drugs

**DOI:** 10.1186/s12935-018-0565-4

**Published:** 2018-05-04

**Authors:** Bodo Haas, Veronika Klinger, Christina Keksel, Verena Bonigut, Daniela Kiefer, Julia Caspers, Julia Walther, Maria Wos-Maganga, Sandra Weickhardt, Gabriele Röhn, Marco Timmer, Roland Frötschl, Niels Eckstein

**Affiliations:** 1 Federal Institute for Drugs and Medical Devices, Kurt-Georg-Kiesinger-Allee 3, 53175 Bonn, Germany; 2 Institute of Pharmacy, University of Bonn, 53121 Bonn, Germany; 3 Applied Pharmacy, University of Applied Sciences Kaiserslautern, Campus Pirmasens, Carl-Schurz-Str. 10-16, 66953 Pirmasens, Germany; 4 Faculty of Applied Natural Sciences, Cologne University of Applied Sciences, Kaiser-Wilhelm-Allee, 51368 Leverkusen, Germany; 5 Department of General Neurosurgery, Center for Neurosurgery, University Hospital Cologne, 50937 Cologne, Germany

**Keywords:** Glioblastoma, Drug resistance, PI3K, Temozolomide, Cisplatin

## Abstract

**Background:**

Intrinsic chemoresistance of glioblastoma (GBM) is frequently owed to activation of the PI3K and MEK/ERK pathways. These signaling cascades are tightly interconnected however the quantitative contribution of both to intrinsic resistance is still not clear. Here, we aimed at determining the activation status of these pathways in human GBM biopsies and cells and investigating the quantitative impact of both pathways to chemoresistance.

**Methods:**

Receptor tyrosine kinase (RTK) pathways in temozolomide (TMZ) treatment naive or TMZ resistant human GBM biopsies and GBM cells were investigated by proteome profiling and immunoblotting of a subset of proteins. Resistance to drugs and RTK pathway inhibitors was assessed by MTT assays. Apoptotic rates were determined by Annexin V staining and DNA damage with comet assays and immunoblotting.

**Results:**

We analyzed activation of RTK pathways by proteome profiling of tumor samples of patients which were diagnosed a secondary GBM and underwent surgery and patients which underwent a second surgery after TMZ treatment due to recurrence of the tumor. We observed substantial activation of the PI3K and MEK/ERK pathways in both groups. However, AKT and CREB phosphorylation was reduced in biopsies of resistant tumors while ERK phosphorylation remained unchanged. Subsequent proteome profiling revealed that multiple RTKs and downstream targets are also activated in three GBM cell lines. We then systematically describe a mechanism of resistance of GBM cell lines and human primary GBM cells to the alkylating drugs TMZ and cisplatin. No specific inhibitor of the upstream RTKs sensitized cells to drug treatment. In contrast, we were able to restore sensitivity to TMZ and cisplatin by inhibiting PI3K in all cell lines and in human primary GBM cells. Interestingly, an opposite effect was observed when we inhibited the MEK/ERK signaling cascade with two different inhibitors.

**Conclusions:**

Temozolomide treatment naive and TMZ resistant GBM biopsies show a distinct activation pattern of the MEK/ERK and PI3K signaling cascades indicating a role of these pathways in resistance development. Both pathways are also activated in GBM cell lines, however, only the PI3K pathway seems to play a crucial role in resistance to alkylating agents and might serve as drug target for chemosensitization.

**Electronic supplementary material:**

The online version of this article (10.1186/s12935-018-0565-4) contains supplementary material, which is available to authorized users.

## Background

Gliomas are divided into four grades with grade IV glioblastoma multiforme (GBM) as the most aggressive form with highest incidence [1, 2]. The high aggressive potential is revealed by a median survival of 14.6 months under therapy, the 5-year survival rate only is at 5.1% [3–5]. The vast majority of GBM occur de novo (primary GBM) while only 5% develop from lower grade II–III astrocytoma forming secondary GBM. Secondary and primary GBM can hardly be distinguished histopathologically but differ from mutations e.g. in the isocitrate dehydrogenase 1 (*IDH1*) gene. While secondary GBM are largely *IDH1* mutant, primary GBM carry the *IDH1* wild-type gene. *IDH1* mutations have been shown to be an independent positive prognostic marker of patient survival [1, 6, 7]. Another important predictor of response to therapy involves *O*^6^-methylguanine-DNA-methyltransferase (*MGMT*) promoter methylation. There is growing evidence that the combination of *IDH1* mutation and *MGMT* promoter methylation is associated with longer survival under therapy [8, 9]. Three main difficulties aggravate GBM therapy. (i) GBM cells spread as single cells rather than building an encapsulated, operable tumor. This infiltrative growth is making GBM virtually incurable by surgery alone. (ii) GBM cells are beyond the blood brain barrier, making them usually unreachable for anti-cancer drugs with affinity to *P*-glycoprotein (*P*-gp) or multi-drug resistance proteins (MRPs) [10]. (iii) GBM cells are intrinsically resistant to chemo- and radiotherapy [11]. This may be owed to frequently mutated tumor suppressor genes known to mediate chemoresistance after loss of function like P53, retinoblastoma protein (RB) and phosphatase and tensin homolog (PTEN) and alterations in receptor tyrosine kinase (RTK) signaling pathways [12, 13]. RTKs such as epidermal growth factor receptor (EGF-R), platelet-derived growth factor receptor (PDGF-R) and insulin-like growth factor 1 receptor (IGF1-R) involved in proliferation, survival and invasiveness of GBM are frequently altered and over-activated [14]. GBM is treated with adjuvant radiotherapy after surgery, while chemotherapy for a long time was not a primary option. In fact, only one chemotherapy-regime results in a significant overall survival benefit: adjuvant temozolomide (TMZ) following radiotherapy [3, 4].

The phosphatidylinositide 3-kinase (PI3K) pathway is a complex, multi-armed signaling network, mediating proliferation, differentiation, migration, metabolism, and survival [15]. Prevention of apoptosis mediated by aberrant PI3K activation or deregulated upstream RTKs is observed in many drug-resistant tumors [16, 17]. The PI3K pathway is frequently over-activated in GBM due to increased RTK signaling or loss of PTEN function [18–23]. In addition, activation of the PI3K pathway in GBM has been correlated with adverse clinical outcome [24, 25]. On the other hand, it has been reported that PTEN and AKT (protein kinase B, PKB) activation status of GBM is irrelevant for prognostic development while activation of extracellular signal-regulated kinase (ERK) is associated with poor patient survival [26, 27]. Mutations in genes of the RAS family of small GTPases commonly observed in many tumor entities are rare for GBM [28]. However, over-activation of the RAS pathway via upstream RTKs or homozygous deletions of the negative RAS regulator neurofibromin 1 (NF1) are common [23]. It has been demonstrated that inhibition of RAS by several miRNAs is able to enhance TMZ-induced apoptosis in GBM cell lines [29, 30]. Mitogen-activated protein kinase kinase (MAPKK/MEK) inhibition however only reduced growth of a small subset of NF1-deficient GBM cell lines [31] and the multi-kinase inhibitor sorafenib failed to enhance radio- and chemosensitivity of GBM cell lines [32].

An array of reports from the literature suggests that inhibition of aberrant PI3K pathway activation may serve to reverse acquired multi-drug resistance in human tumors [33–35]. Consequently, the PI3K pathway has emerged as a promising treatment modality, with the potential to act synergistically with chemotherapy [36–39]. To this end, it has been shown that inhibition of PI3K by small molecules can sensitize tumor cells to various DNA damaging drugs by induction of apoptosis [40–44]. In contrast, another study has shown that inhibition of PI3K in primary GBM stem cells and differentiated GBM cells did not substantially chemosensitize cells to drug treatment [45]. Despite encouraging pre-clinical results, positive clinical outcome with PI3K inhibitors in GBM is limited. Thus, solely inhibiting the PI3K arm of RTK signaling cascades as the magic bullet appears questionable. Several reasons for the lack of efficacy are discussed [46]. Among others blocking PI3K and in particular downstream targets such as AKT and mammalian target of rapamycin (mTOR) counter regulatory activate RTKs and downstream signaling such as the RAS cascade allowing the cell to bypass PI3K/AKT blockade [47–50].

Therefore, we aimed at further disentangling the quantitative contribution of the PI3K/AKT and MEK/ERK pathways to intrinsic resistance to alkylating drugs in GBM. We investigate the activation status of RTK pathways in GBM tumor biopsies and GBM cell lines and subsequently elaborate the molecular mechanism of resistance to the DNA-alkylating anti-cancer drugs cisplatin and TMZ in three *PTEN* mutated GBM cell lines and primary GBM cells derived from tumor biopsies.

## Methods

### Materials

Temozolomide (Temodal^®^), LY294002, U0126, AG1024, AG1296, erlotinib (Tarceva^®^), wortmannin, U0126, and PD98059 were purchased from Sigma-Aldrich.

### Tumor samples

Human tissue specimens of secondary GBM were taken from the tumor tissue bank of the Clinic of Neurosurgery, University Hospital Cologne. The collection of samples was approved by the University’s Institutional Ethical Board. Informed consent of the patients was obtained according to the Helsinki declaration of ethical requirements.

Tissue samples were obtained directly from neurosurgery between 1990 and 2015, immediately snap-frozen in liquid nitrogen and stored at − 80 °C. 10 µm cryostat sections were taken from each sample, stained with HE for histological examination by a neuropathologist in order to assure that representative tissues were used for biochemical analysis. Histopathological diagnosis and grading was based on the WHO classification 2007 [51]. Mutation analysis was performed as previously described [52].

### Cell culture

U87-MG, U251-MG, and U373-MG (Uppsala) GBM cell lines were obtained by Sigma-Aldrich (HPA Culture Collections) in 2015 and not used beyond passage 20. U251 Cells were cultivated in RPMI 1640 (Biochrom) containing 10% FCS (Biochrome), 100 IU/ml penicillin, and 100 µg/ml streptomycin (Biowest). All other cells and cell lines were cultivated in DMEM low Glucose (Biowest) containing 10% FCS, 100 IU/ml penicillin, and 100 µg/ml streptomycin. Primary GBM cells derived from a primary GBM tumor biopsy were obtained from the University Hospital Cologne. *IDH1* mutation and *MGMT* promoter methylation status [52] of all primary cells and cell lines used is displayed in Table 1.

**Table 1:** *IDH1* mutation and *MGMT* promoter methylation status of GBM cell lines and primary cells

Cells	IDH1	MGMT promoter
U251-MG	Wild-type	Methylated
U373-MG	Wild-type	Methylated
U87-MG	Wild-type	Methylated
Primary culture	Wild-type	Methylated

### Proteome profiling

The functional status of signaling pathways was measured by Proteome Profiler™ human phospho-RTK, human apoptosis and human phospho-MAPK antibody arrays (R&D Systems). Tissue samples were disrupted with ice cold lysis buffer and a tissue homogenizer. Cells were lysed after incubation with respective inhibitors and TMZ by addition of ice cold lysis buffer to PBS washed culture plates. After centrifugation the protein content of the supernatant was determined and 200–300 µg of total protein was used for proteome profiling according to the manufactures protocol and as described previously [53].

### Western blot analysis

For immunoblotting standard procedures using the following antibodies were used as previously described [53, 54]. Anti-EGF-R (AF-231), anti-IGF-R (AF-305-NA), anti-PDGF-Rβ (AF-385) combined with donkey anti-goat IgG-HRP (HAF 109; all R&D systems). Anti-MRP-2 (M2 III-6), Anti-P-gp (D-11; both Santa Cruz Biotechnology) combined with goat anti-mouse IgG (H + L)-HRP (Thermo Scientific). Anti-P53-phospho-S15 (A7180; Assay Biotechnology Company), P21 (C-19, Santa Cruz Biotechnology) combined with goat anti-rabbit IgG (H + L)-HRP (Thermo Scientific). P53-HRP (DO-1) and β-Actin-HRP (C-4; both Santa Cruz Biotechnology). Immunoblots were developed with the enhanced chemoluminescence system (Amersham Biosciences).

### MTT assay

For MTT assays we followed a published protocol [55]. Briefly, cell survival after exposure to cisplatin or TMZ and in the presence of signaling inhibitors was determined by MTT assays. Signaling inhibitors 1 µM AG1024, 10 ng/ml AG1296, 3 µM Erlotinib, 2–20 µM LY294002, 30 nM Wortmannin, 5–20 µM U0126, and 5–20 µM PD98059 were added to culture medium prior to addition of cisplatin or TMZ for 72 h.

### Annexin V apoptosis assay

For apoptosis measurements the BD Pharmingen™ FITC Annexin V Apoptosis Detection Kit (BD Biosciences) was used according to the manufacturer’s protocol. Briefly, 2.5 × 10^5^ cells were seeded into 6-well plates and incubated at 37 °C and 5% CO_2_ overnight. After compound treatment for 72 h, cells were trypsinized and centrifuged for 4 min at 1500×*g*. Medium supernatant was removed and cells were resuspended in 500 µl binding buffer. 5 µl PI and 5 µl Annexin V-FITC were mixed with 100 µl of cells in binding buffer. After 15 min of incubation on ice, samples were analyzed on a flow cytometry (FACSCalibur™, BD Bioscience).

### Alkaline comet assay

1.25 × 10^5^ cells were seeded into 6-well plates. After 24 h cells were treated with 20 µM LY294002 and TMZ for 1 h and washed with PBS. Cells were released from the culture plate by Accutase. Comet assays were performed as previously described [56]. For analysis of comets, 50 cells were selected randomly by image analyzing (Comet Assay IV, Perceptive instruments) using a Zeiss Axioskop 2 (Carl Zeiss, Jena).

### Data analysis and statistical methods

Concentration effect curves were fitted to data points by nonlinear regression analysis using the four-parameter logistic equation (GraphPad™ Prism). Statistical differences between two groups were determined by unpaired 2-tailed Student’s *t* test. Comparisons among several groups were performed by ANOVA followed by Turkey post hoc test. Data are presented as mean ± SEM.

## Results

Glioblastoma multiforme are reported to have various deregulated signaling pathways which drive resistance towards radio- and chemotherapy [23, 57]. In order to investigate the activation of RTK pathways in vivo, we performed human proteome profiler arrays with a subset of proteins of tumor biopsies of four patients, which were diagnosed a secondary GBM and underwent surgery before TMZ treatment and of four patients which underwent a second surgery later on after TMZ treatment due to recurrence of the tumor. Due to time of sampling of the biopsies between years 1990 and 2015, grading of tumors was based on histopathological analysis and clinical observations according to the 2007 WHO classification [51]. Additional *IDH1* mutation analysis revealed that all GBM samples with one exception (P8) were *IDH1* mutant (Table 2) and could therefore also be classified as secondary GBM according to the recent 2016 WHO classification [1]. P8 was clinically judged as secondary GBM due to a previously diagnosed precursor lesion (low-grade glioma) and was therefore included in the analysis despite lack of *IDH1* mutation.

**Table 2:** *IDH1* mutation status of secondary GBM samples

Patient ID	Treatment	IDH1
P1	–	Mutated
P2	–	Mutated
P3	–	Mutated
P4	–	Mutated
P5	TMZ	Mutated
P6	TMZ	Mutated
P7	TMZ	Mutated
P8	TMZ	Wild-type

**Fig. 1:**
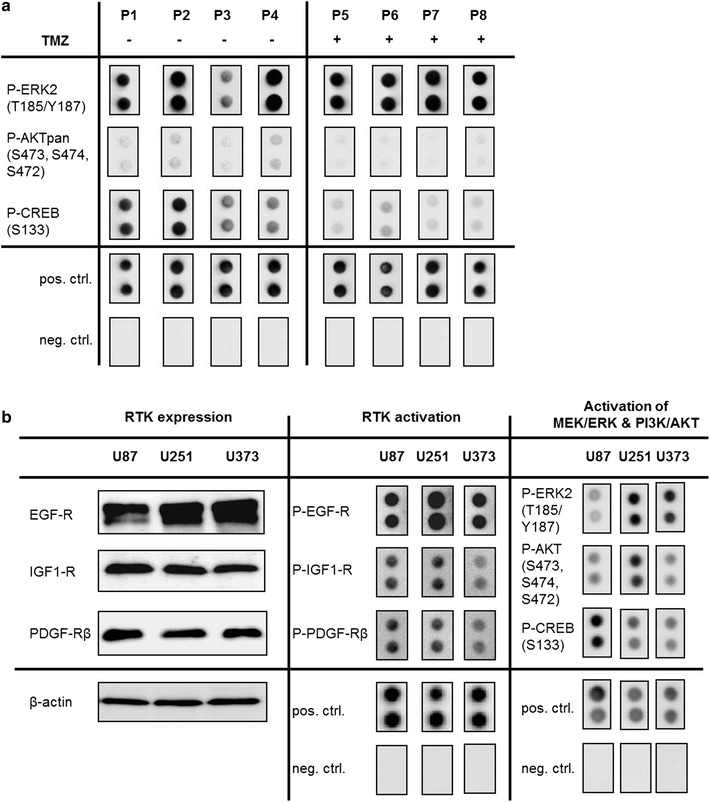
The MEK/ERK and PI3K pathways are activated in GBM in vivo and in vitro. **a** Human proteome-profiler phospho-MAPkinase arrays of GBM tumor biopsies before and after TMZ treatment. Shown are biopsies derived from patients, who were newly diagnosed a secondary GBM and underwent surgery before TMZ treatment (P1–P4) and of patients which underwent a second surgery after TMZ treatment due to recurrence of the tumor (P5–P8). Phosphorylated ERK2 (T185/Y187, AKTpan (S473, S474, S472) and CREB (S133) were detected in all biopsies. **b** Western blotting reveals that EGF-R, IGF1-R, and PDGF-Rβ RTKs are constitutively overexpressed. β-actin Western blot was performed to control for loading (left panel). Human proteome-profiler phospho-antibody arrays were used to assess the activation status of RTKs (middle panel) and downstream targets ERK2 (T185/Y187), AKTpan (S473, S474, S472) and CREB (S133) (right panel)

We found that several kinases were phosphorylated in all eight patients (Additional file 1: Figure S1a). Strikingly, there was a trend (*P* < 0.05) towards reduced phosphorylation of AKT and its downstream target cAMP response element-binding protein (CREB) in GBM of patients resistant to TMZ therapy while ERK activation remained unchanged on a high level (Fig. 1a and Additional file 1: Figure S1a) indicating a role of these kinase in resistance development. It is also interesting to note that several apoptotic proteins were expressed in GBM biopsies but only small differences were observed between untreated and TMZ treated and resistant patients (Additional file 1: Figure S1b). Only reduction in pro-caspase-3 expression in TMZ treated patients reached statistical significance (*P* < 0.05).

This led us to further elucidate these findings in vitro. As *IDH1* mutant cell lines are rare we focused our study on *IDH1* wild-type GBM cell lines and cells (Table 1). GBM cells are reported to be intrinsically resistant to anti-cancer drugs and different sensitivities to TMZ are reported in the literature. Thus, we initially characterized RTK signaling pathway expression and activation in U87, U251, and U373 GBM cell lines. EGF-R, IGF1-R, and PDGF-Rβ RTKs are overexpressed (Fig. 1b, left panel) and activated (Fig. 1b, middle panel) in all GBM cell lines. All of these growth factor receptors converge on MEK/ERK and PI3K pathway activation, which is reflected by phosphorylation of ERK, AKT and the downstream target CREB (Fig. 1b, right panel). Thus, as observed in vivo the MEK/ERK and PI3K pathways are also active in GBM cells and might be responsible for drug resistance.

Yet TMZ is the only substance for which a statistically significant and clinically relevant benefit in both, overall survival and progression free survival was demonstrated. Thus, TMZ was approved by both, EMA and US-FDA, for orally active cytostatic therapy of GBM. However, it is very difficult to handle in the laboratory setting as it must be given to cell culture assays in concentrations as high as or even beyond its solubility. In addition, TMZ is a prodrug with a highly complex pharmacokinetic bioactivation pattern and intracellular distribution. Thus, as many other groups, we also used cisplatin as a model substance with a similar molecular mechanism of action: both substances are DNA-alkylating agents.

**Fig. 2:**
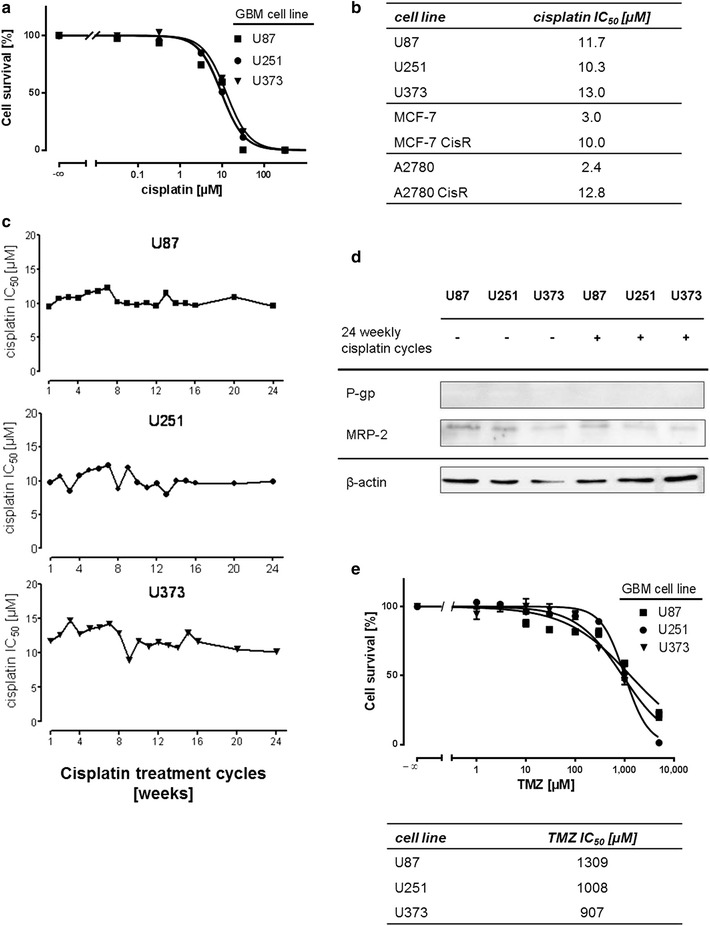
GBM cell lines show high intrinsic tolerance to alkylating cytostatics cisplatin and TMZ. **a** Cisplatin resistance status of U87, U251, and U373 cell lines was assessed by MTT assays (n = 4). **b** Intrinsic resistance of GBM cell lines is demonstrated in comparison to cell pairs of non-resistant and cells with acquired resistance. GBM cell line IC_50_ concentrations of cisplatin parallel the ones of resistant MCF-7 breast-, and A2780 ovarian cancer cell lines (determined by MTT assays; n = 3). **c** GBM cell lines were treated by weekly cycles of cisplatin and IC_50_ values were assayed after each treatment cycle in MTT assays. IC_50_ values were obtained from sigmoidal concentration–response curves and dotted in a time-dependent manner. **d** Western blots showing MRP-2 and *P*-gp expression in GBM cell lines untreated and after 24 cycles of weekly intermittent cisplatin exposure. β-actin Western blot was performed to control for loading. **e** TMZ resistance status in U87, U251 and U373 cells was assessed by MTT assays (upper graph; n ≥ 6). The respective IC_50_ values for each cell line are shown in the table below the graph

We first characterized the status of cisplatin resistance in GBM cell lines U87, U251, and U373 by MTT cell survival assays (Fig. 2a). Intrinsic resistance of the cells is also demonstrated in Fig. 2b, where cisplatin IC_50_ concentrations of GBM cell lines are compared to non-resistant and cisplatin resistant breast and ovarian cancer cell lines as previously reported by us [53, 55]. GBM cell lines showed cisplatin tolerance comparable to cells with acquired cisplatin resistance and exhibit an average IC_50_ concentration of 10 µM, representing about threefold the concentration maximally achieved in plasma of patients treated with cisplatin. We then wished to investigate, whether the intrinsic resistance in GBM cells can be strengthened by long-term intermittent application of cisplatin in a way the drug is administered to patients. Therefore, we used a 6-month protocol of weekly intermittent cisplatin exposure, which we have used previously to generate cisplatin-resistant breast- and ovarian cancer cells [53, 55]. Interestingly, no gain in cisplatin tolerance could be observed in either of the three cell lines (Fig. 2c). We conclude that no intracellular pathway alteration emerged after 6 months of weekly intermittent cisplatin treatment. In addition, we assumed that no transport protein expression has occurred over the 24 weekly treatment cycles, which is also demonstrated by low to undetectable *P*-gp and MRP-2 expression (Fig. 2d). In addition, GBM cell lines displayed high resistance towards TMZ (Fig. 2e) with calculated IC_50_ values at around 1 mM TMZ (table in Fig. 2e). We were not able to determine the bottom of the concentration response curve for U87 and U373 cells in MTT assays at feasible TMZ concentrations. To determine IC_50_ values curves were extrapolated to zero.

**Fig. 3:**
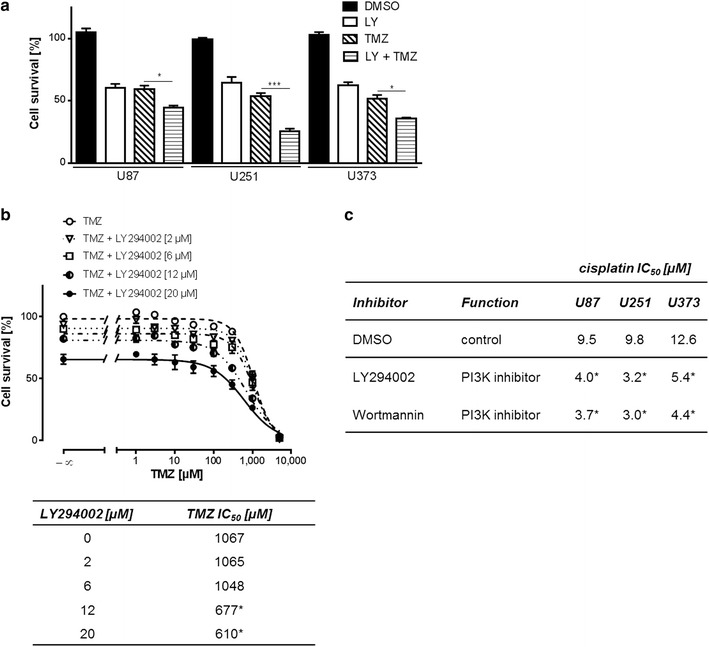
PI3K as a key component of TMZ resistance in GBM cells. **a** PI3K inhibition (20 µM LY294002) sensitizes all GBM cells to TMZ (1000 µM) (n = 3). **b** MTT concentration response curve showing concentration dependency of sensitization by LY294002 in U251 cells (upper graph). Respective TMZ IC_50_ values at increasing LY294002 concentrations are displayed in the table below the graph (n ≥ 3). **c** Both, LY294002 (5 µM) and Wortmannin (30 nM), sensitize GBM cell lines to cisplatin. Presented IC_50_ values were derived from MTT assays (n = 3). *P < 0.05, ***P < 0.001

To inhibit RTKs and downstream signaling pathways, we used small molecule inhibitors, each of them targeting specifically one protein of the activated network. However, no specific inhibitor of any of the RTKs was able to overcome resistance (Additional file 2: Figure S2). Due to their relevance in drug resistance, we used two structurally different substances to inhibit MEK/ERK, and PI3K/AKT signaling, respectively. All cell lines under investigation show pronounced PI3K/AKT pathway activation (Fig. 1b), which one would expect to be caused by *PTEN* mutation. Therefore, we tested initially if inhibition of PI3K mediated AKT phosphorylation was capable of sensitizing the cell lines to TMZ. Inhibition of this survival pathway with the PI3K inhibitor LY294002 triggered capability of the cells to apoptotic stimuli like TMZ (Fig. 3a). Reduction of AKT phosphorylation after LY294002 treatment for 72 h in U251 cells is shown in Additional file 3: Figure S3. The sensitizing effect of LY294002 was most pronounced in U251 cells. Therefore, in further experiments with TMZ we focused on this cell line. In order to quantitatively characterize the concentration response relationship we recorded concentration response curves with increasing LY294002 and TMZ concentrations (Fig. 3b). We observed a systematic left shift of the TMZ concentration response curve at increasing LY294002 concentrations becoming significant at 12 µM LY294002 (table in Fig. 3b). LY294002 and wortmannin, another PI3K inhibitor, also significantly left-shifted cisplatin concentration response curves in all cell lines. Cisplatin IC_50_ values derived from respective curves are displayed in Fig. 3c.

**Fig. 4:**
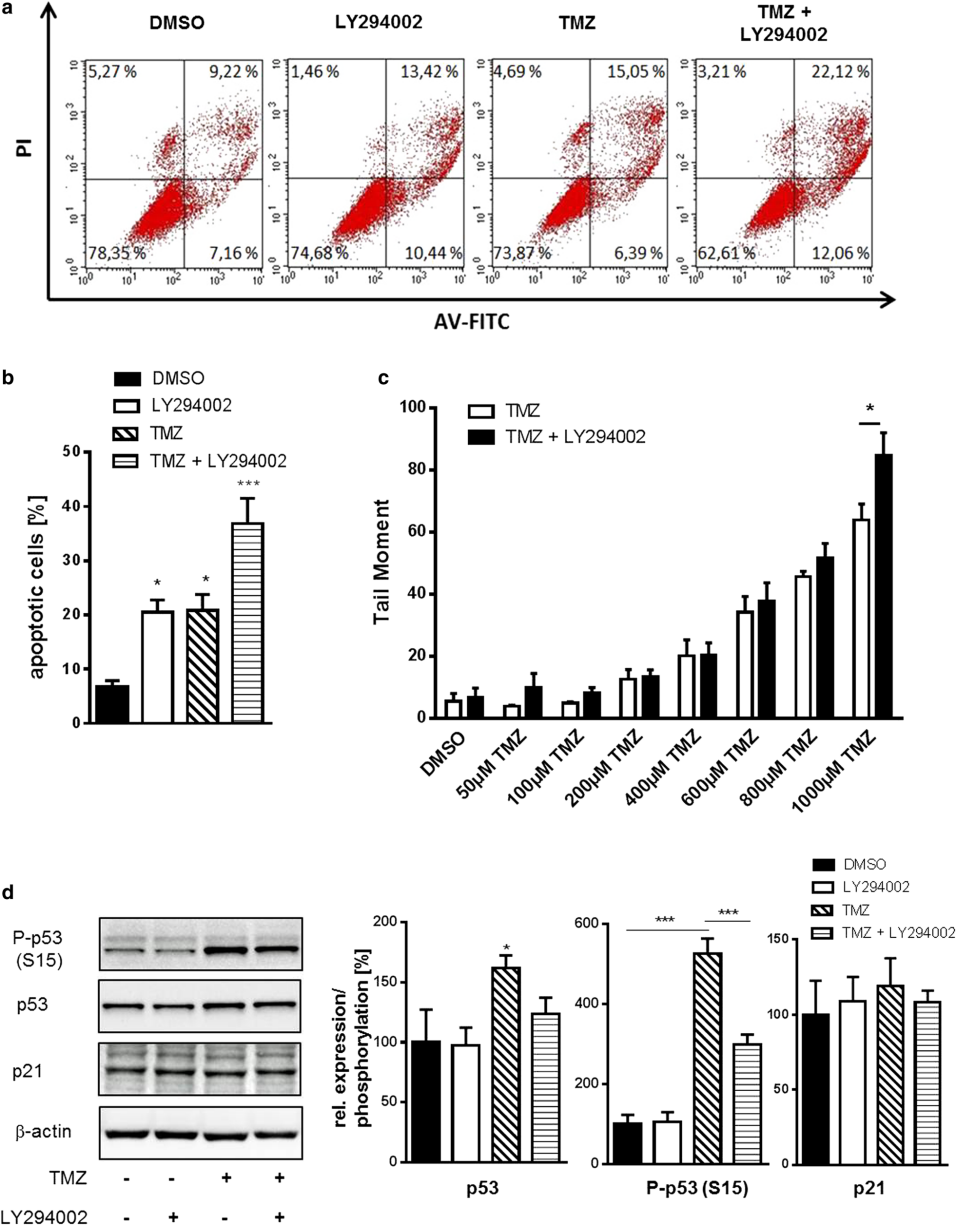
LY290042 increases apoptosis and DNA damage of TMZ treated U251 cells. **a** Representative FACS blots of U251 cells treated with either vehicle (DMSO), TMZ (1000 µM), LY294002 (20 µM) or the combination for 72 h. Annexin V positive cells were regarded as apoptotic cells. **b** Quantification of apoptotic cells from Annexin V assays (n = 5). **c** DNA damage was assayed by comet assay and is displayed as tail moment in U251 cells treated with LY290042, TMZ or vehicle (DMSO) for 1 h as indicated. **d** Representative Western blots showing P–P53 (S15), P53 and P21 expression in U251 cells treated with LY290042 (20 µM), TMZ (1000 µM) or vehicle (DMSO) for 24 h as indicated. β-actin Western blot was performed to control for loading (left panels). Densitometric analysis of the respective (phospho-) proteins relative to β-actin (right bars, n = 3). *P < 0.05, ***P < 0.001

Annexin V apoptosis assays of U251 cells confirmed an increase in apoptotic rates after combination treatment when compared to TMZ or LY294002 treatment alone (Fig. 4a, b). Furthermore, combination treatment also resulted in significantly increased DNA damage as determined by comet assays (Fig. 4c). These data confirm findings from Westhoff et al. [42] who have shown that inhibition of PI3K and DNA-dependent protein kinase (DNA-PK), a kinase involved in DNA repair and also inhibited by LY294002, sensitize GBM cells to cytostatic drugs by interfering with DNA damage repair. In order to gain insight into the molecular mechanisms we performed proteome profiler arrays with cell lysates from TMZ and LY294002 treated U251 cells. TMZ induced phosphorylation of AKT and P53 and expression of a variety of apoptotic and anti-apoptotic proteins. Notably, concomitant LY294002 treatment abolished TMZ-induced effects such as P53 phosphorylation or claspin expression indicating that DNA damage repair is disturbed by LY294002 (Additional file 3: Figure S3). Western blotting of P53 and phosphorylated P53 (S15) confirmed these findings. Interestingly despite P53 activation, P21 protein abundance was not affected by TMZ or LY294002 treatment (Fig. 4d).

**Fig. 5:**
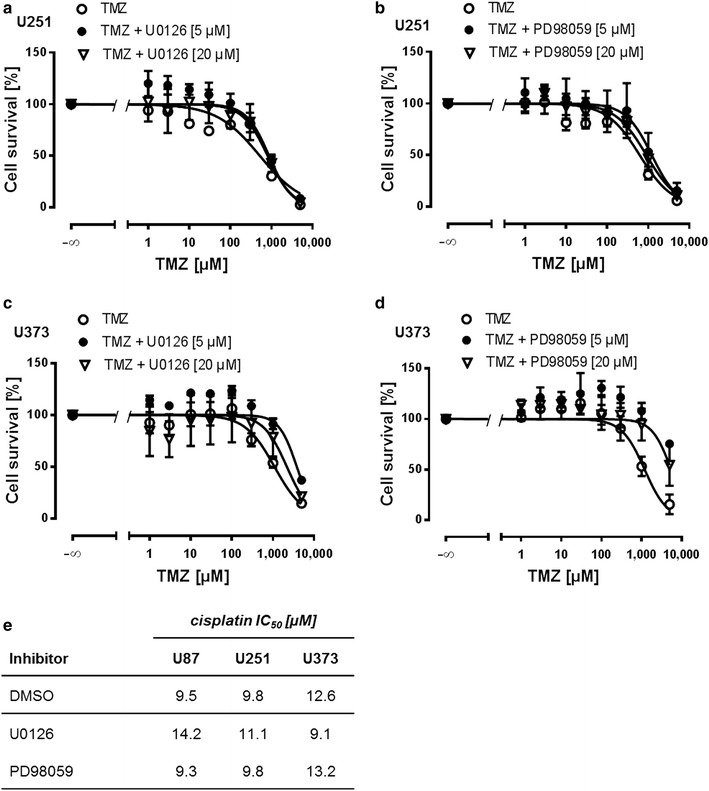
MEK inhibition negatively impacts on intrinsic chemoresistance. MTT assays showing that **a** U0126 (5 and 20 µM) and **b** PD98059 (5 and 20 µM) desensitize U251 cells for TMZ treatment (n = 3). **c** U0126 (5 and 20 µM) and **d** PD98059 (5 and 20 µM) lead to a strong right shift of TMZ concentration response curves in U373 cells (n = 3). **e** U0126 (10 µM) and PD98059 (20 µM) do not sensitize GBM cell lines to cisplatin. Presented IC_50_ values were derived from MTT assays (n = 3)

In a next step we targeted MEK with two specific inhibitors, U0126 and PD98059, respectively. Cell lines U373 and U251 have been reported to be both NF1-deficient and to display different sensitivities for MEK inhibition [31]. Concentration response experiments with TMZ in both cell lines revealed no significant left shift of the curves after concomitant U0126 or PD98059 treatment at 5 and 20 µM, respectively. Conversely, MEK inhibition even tended to desensitize cells for TMZ treatment and this effect was more pronounced in U373 cells which have been reported to be more sensitive to MEK inhibition (Fig. 5a–d) [31]. This might be owed to reduced proliferation velocity and subsequent reduced ability of TMZ to alkylate DNA during mitosis. Indeed, U0126 and PD98059, respectively, almost completely block proliferation of U373 and U251 cells (Additional file 4: Figure S4). Similar results were obtained for cisplatin in all other cell lines (Fig. 5e). These data indicate that the MEK/ERK pathway is negatively associated to GBM intrinsic chemoresistance.

**Fig. 6:**
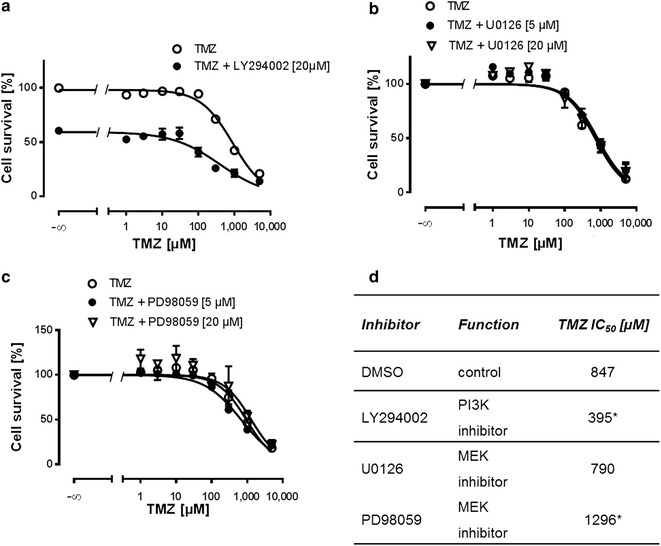
PI3K but not MEK inhibition sensitizes human primary GBM cells to TMZ. **a** PI3K inhibition (20 µM LY294002) leads to a left shift of the TMZ concentration response curve (n = 3). **b** Neither, U0126 (5 and 20 µM; n ≥ 3) **c** nor PD98059 (5 and 20 µM; n ≥ 3) sensitize primary cells to TMZ. **d** Derived mean IC_50_ values for TMZ of respective primary GBM cells treated with LY294002 and MEK inhibitors (20 µM each; n ≥ 3). *P < 0.05

Finally, we asked whether our findings can be translated to a more clinically relevant scenario. Therefore, we isolated human primary GBM cells from a tumor biopsy. In line with our findings in GBM cell lines, inhibition of PI3K also sensitized primary cells to TMZ. In addition, MEK inhibition with U0126 or PD98059 did not affect cellular resistance of these cells (Fig. 6a–d) indicating that inhibition of PI3K signaling to enhance chemotherapy might also be relevant in patients.

## Discussion

An important mechanism of chemoresistance of GBM is an intrinsic high activation status of the PI3K/AKT survival pathway due to frequent mutations in the *PTEN* gene [19–21]. It appears logical that inhibition of this pathway should enhance chemosensitivity and prone cells to apoptosis. Pre-clinical results are encouraging but, clinical evidence of efficacy of inhibitors of the PI3K/AKT pathway is still lacking [46]. Several reasons might be responsible. Targeting the brain beyond the blood brain barrier is often not feasible leading to failure of drug development due to pharmacokinetic reasons. In addition, PI3K is a ubiquitous enzyme and inhibition is frequently associated with adverse events leading to termination of clinical trials for safety reasons despite efficacy. On a molecular level counter regulatory mechanisms involving upregulation of RTKs or the RAS pathway have also been described to be involved in limiting PI3K inhibitor efficacy [47–50]. We found a strong phosphorylation of proteins of the PI3K and MEK/ERK pathways in very rare biopsies of secondary GBM naive to TMZ and resistant to TMZ treatment. Secondary GBM itself do only constitute a very small subgroup of GBM accounting for not more than 5% in total. Thus, to gain these very rare biopsies *before* and *after* TMZ treatment took great effort. Interestingly, phosphorylation of AKT and its downstream effector CREB was reduced in TMZ-resistant tumors indicating that these pathways are deregulated and are probably involved in resistance development while no changes were observed in ERK phosphorylation status. These findings however have to be interpreted with caution as secondary GBM are highly heterogeneous and only a small number of biopsies from eight different patients was analyzed. Thus, it was even more remarkable that significant differences were observed between groups despite the limitations described above. These findings encouraged us to further elaborate the quantitative impact of these pathways on chemoresistance in in vitro experiments. Due to the scarcity of available material, we were not able to obtain secondary GBM cells with an *IDH1* mutation and based our study on *IDH* wild-type cells. Despite histopathological similarities, primary and secondary GBM derive from different precursor cells and are distinct tumor entities [7] what makes translation of our results difficult. In addition, *MGMT* promoter methylation status was only available for cells but not for tumor samples. As both, *IDH1* mutation and MGMT expression, can predict outcome of therapy [8, 9] obtained in vitro results cannot directly be translated to the clinical situation of our tumor sample analysis.

We found that GBM cell lines were highly resistant to cisplatin comparable to resistant breast (MCF-7 CisR) and ovarian cancer cells (A2780 CisR) [53]. IC_50_ values for TMZ were in the range of 1 mM for all cell lines indicating high intrinsic resistance also to TMZ.

Since, PI3K and MEK/ERK signaling cascades operate downstream of membrane-spanning growth-factor receptors, it is intuitive that inhibition of receptors for EGF, IGF-1, and PDGF did not impact on cisplatin resistance. This is in line with clinical data that small molecule RTK inhibitors or monoclonal antibodies designed to target RTKs failed to improve GBM therapy [50, 51]. It has previously been reported that PI3K inhibition sensitizes tumor cells to extrinsic TRAIL-induced apoptosis as well as apoptosis induced by DNA damaging agents [40–44, 58] while another study suggests that PI3K inhibition in GBM stem cells and differentiated primary cells only leads to poor or negative chemosensitization [45]. These discrepancies drove us to investigate these findings in more detail in a quantitative manner and finally—to generalize our findings—in primary human GBM cells. A systematic and statistically significant left shift of concentration response curves and approximately twofold reduction of TMZ and cisplatin IC_50_ values was observed upon PI3K inhibition in all three cell lines and most importantly also in primary cells. Investigating the molecular mechanism of this chemosensitization we found that PI3K inhibition induced DNA damage which is probably mediated by inhibition of DNA repair after TMZ treatment. This is further strengthened by the fact, that TMZ-induced P53 phosphorylation and claspin expression, which both are involved in DNA damage response, were reversed by LY294002 treatment. These data confirm findings from Westhoff et al. [42] who have shown that LY294002 sensitize GBM cells to cytostatic drugs by interfering with DNA damage repair. Nevertheless, it cannot finally be concluded from these experiments if inhibition of DNA repair by LY294002 is solely due to inhibition of PI3K or also inhibition of other members of the PI3K family such as DNA-PK, ataxia-telangiectasia-mutated kinase (ATM) and Rad3-related kinase (ATR). However, data from other groups suggest, that inhibition of ATR/ATM and DNA-PK by LY294002 does not occur at the concentrations tested here (20 µM) and that effects on DNA damage repair are probably mediated by PI3K inhibition [53–55].

We found a negative impact of the MEK/ERK pathway on chemosensitivity upon inhibition with two different inhibitors in all cell lines and primary cells. Both MEK inhibitors led to an almost complete growth arrest. As TMZ is an agent which damages DNA by forming *O*6-alkylguanine [59], a process which requires cell proliferation, it might explain why MEK inhibition was not successful in sensitizing cells to TMZ and cisplatin. These findings are in line with Riedel et al. who have shown that the RAF inhibitor sorafenib reduced proliferation but failed to enhance chemosensitivity of GBM cells [32].

## Conclusion

Taken together, our data indicate that the PI3K pathway is one of the main pathways involved in GBM chemoresistance. Combination of TMZ with PI3K inhibitors might therefore result in clinical benefit for GBM patients when problems of pharmacokinetics and safety of these compounds can be addressed. This is underlined by the fact that several inhibitors of the PI3K/mTOR axis are currently undergoing clinical development for GBM [39]. A further implication of our study is that simply combining an inhibitor of the MEK/ERK signaling cascade with chemotherapy in GBM is probably not a recommendable strategy.

## Additional files

**Additional file 1: Figure S1.** Densitometric analysis of (**a**) human proteome profiler phospho-MAPkinase and (**b**) human apoptosis arrays of GBM tumor biopsies of patients which were newly diagnosed a secondary GBM and underwent surgery before TMZ treatment (n=4) and of patients which underwent a second surgery after TMZ treatment due to recurrence of the tumor (n=4). Phosphorylation was normalized to positive control spots. *P < 0.05, **P < 0.01.

**Additional file 2: Figure S2.** Impact of RTK inhibitors on cisplatin resistance in GBM cell lines. RTK inhibitors erlotinib (3 µM), AG1024 (1 µM), and AG1296 (10 ng/mL) do not sensitize GBM cell lines to cisplatin. Presented IC_50_ values were derived from MTT assays (n=3).

**Additional file 3: Figure S3.** Human proteome-profiler (**a**) phospho-MAPKkinase and (**b**) apoptosis arrays of U251 cells treated with LY290042 (20 µM), TMZ (1000 µM) or vehicle (DMSO) for 72 hours.

**Additional file 4: Figure S4.** MEK inhibition blocks cell proliferation of U251 and U373 cells. (**a**) U251 cells and (**b**) U373 cells were treated with vehicle (DMSO), U0126 or PD98059 as indicated (n=3). Cells were trypsinized at 24, 48 and 72 hours post treatment and counted. Cell number at time point 0 hours was set to 1.

## Abbreviations

AKT: protein kinase B (PKB)
ATM: ataxia-telangiectasia-mutated kinase
ATR: Rad3-related kinase
GBM: glioblastoma multiforme
CREB: cAMP response element-binding protein
DNA-PK: DNA-dependent protein kinase
EGF-R: epidermal growth factor receptor
ERK: extracellular signal-regulated kinase
IDH: isocitrate dehydrogenase
IGF1-R: insulin-like growth factor 1 receptor
MEK: mitogen-activated protein kinase kinase (MAPKK)
MGMT: *O*^6^-methylguanine-DNA-methyltransferase
MRP: multi-drug resistance proteins
mTOR: mammalian target of rapamycin
NF1: neurofibromin 1
PDGF-R: platelet-derived growth factor receptor
*P*-gp: *P*-glycoprotein
PI3K: phosphatidylinositide 3-kinase
PTEN: phosphatase and tensin homolog
RB: retinoblastoma protein
RTK: receptor tyrosine kinase
TMZ: temozolomide
