# Supplementary figures and images for: Inhibition of the PI3K but not the MEK/ERK pathway sensitizes human glioma cells to alkylating drugs
Source: Cancer Cell Int. 2018 May 4;18:69. doi: 10.1186/s12935-018-0565-4 (PMC5935937; doi:10.1186/s12935-018-0565-4)

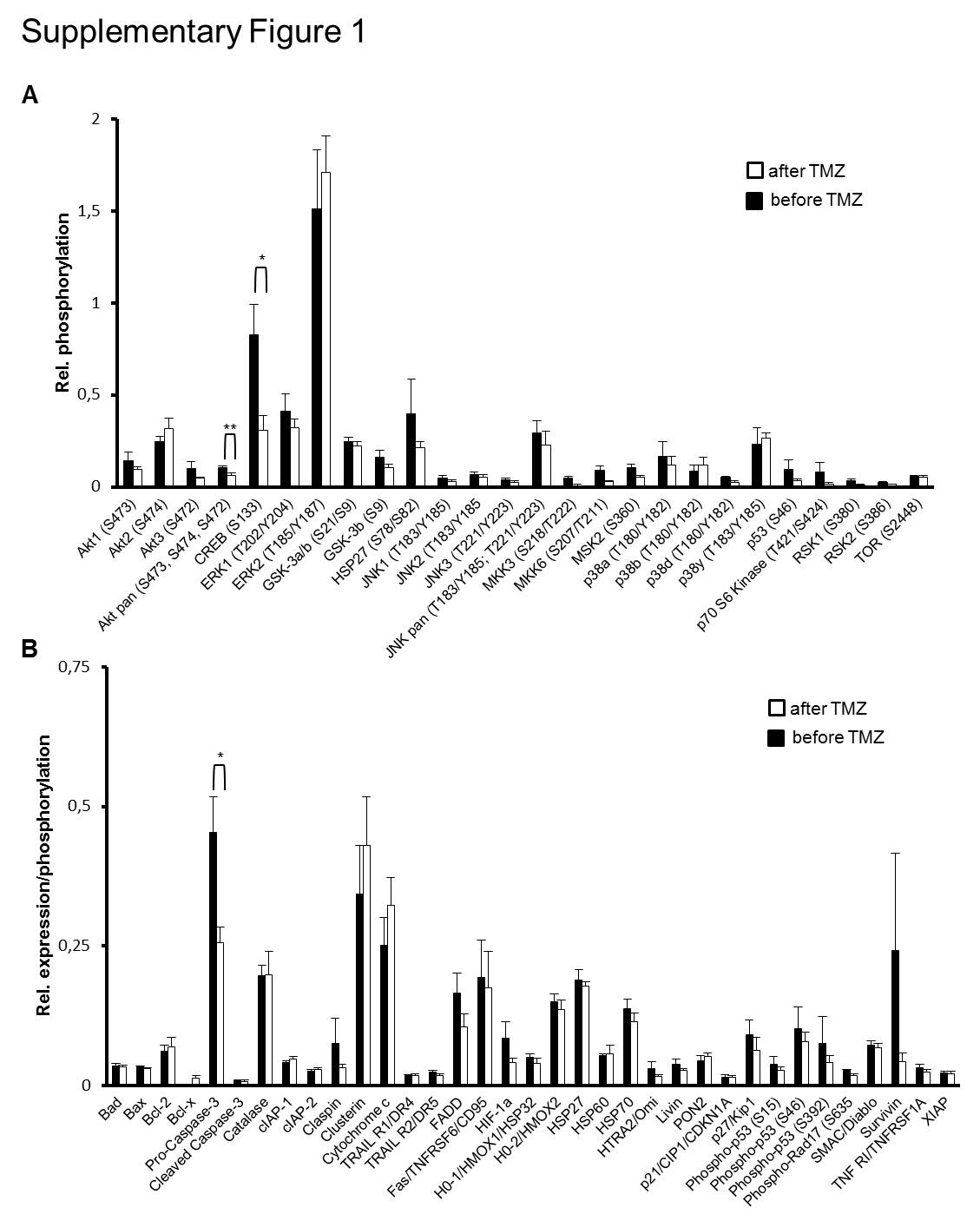

Supplement: Supplementary file 1 — Additional file 1: Figure S1. Densitometric analysis of (a) human proteome profiler phospho-MAPkinase and (b) human apoptosis arrays of GBM tumor biopsies of patients which were newly diagnosed a secondary GBM and underwent surgery before TMZ treatment (n=4) and of patients which underwent a second surgery after TMZ treatment due to recurrence of the tumor (n=4). Phosphorylation was normalized to positive control spots. *P < 0.05, **P < 0.01. [file 12935_2018_565_MOESM1_ESM.jpg]

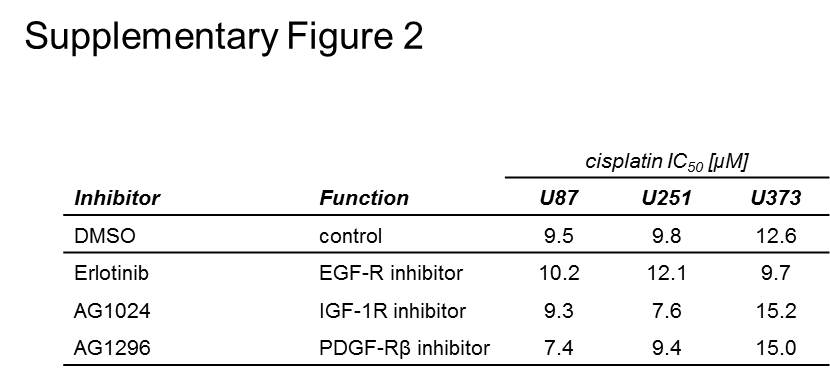

Supplement: Supplementary file 2 — Additional file 2: Figure S2. Impact of RTK inhibitors on cisplatin resistance in GBM cell lines. RTK inhibitors erlotinib (3 µM), AG1024 (1 µM), and AG1296 (10 ng/mL) do not sensitize GBM cell lines to cisplatin. Presented IC50 values were derived from MTT assays (n=3). [file 12935_2018_565_MOESM2_ESM.jpg]

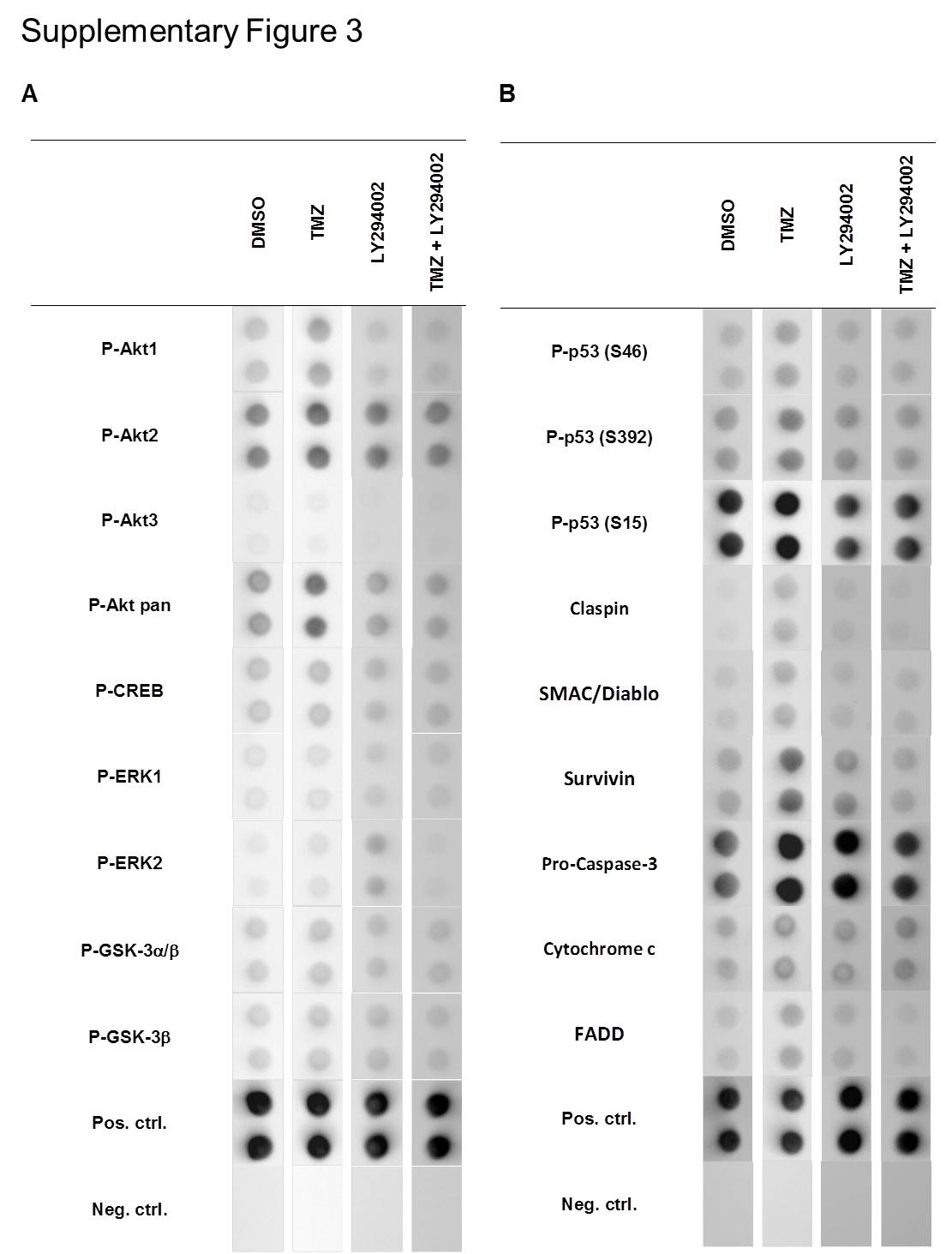

Supplement: Supplementary file 3 — Additional file 3: Figure S3. Human proteome-profiler (a) phospho-MAPKkinase and (b) apoptosis arrays of U251 cells treated with LY290042 (20 µM), TMZ (1000 µM) or vehicle (DMSO) for 72 hours. [file 12935_2018_565_MOESM3_ESM.jpg]

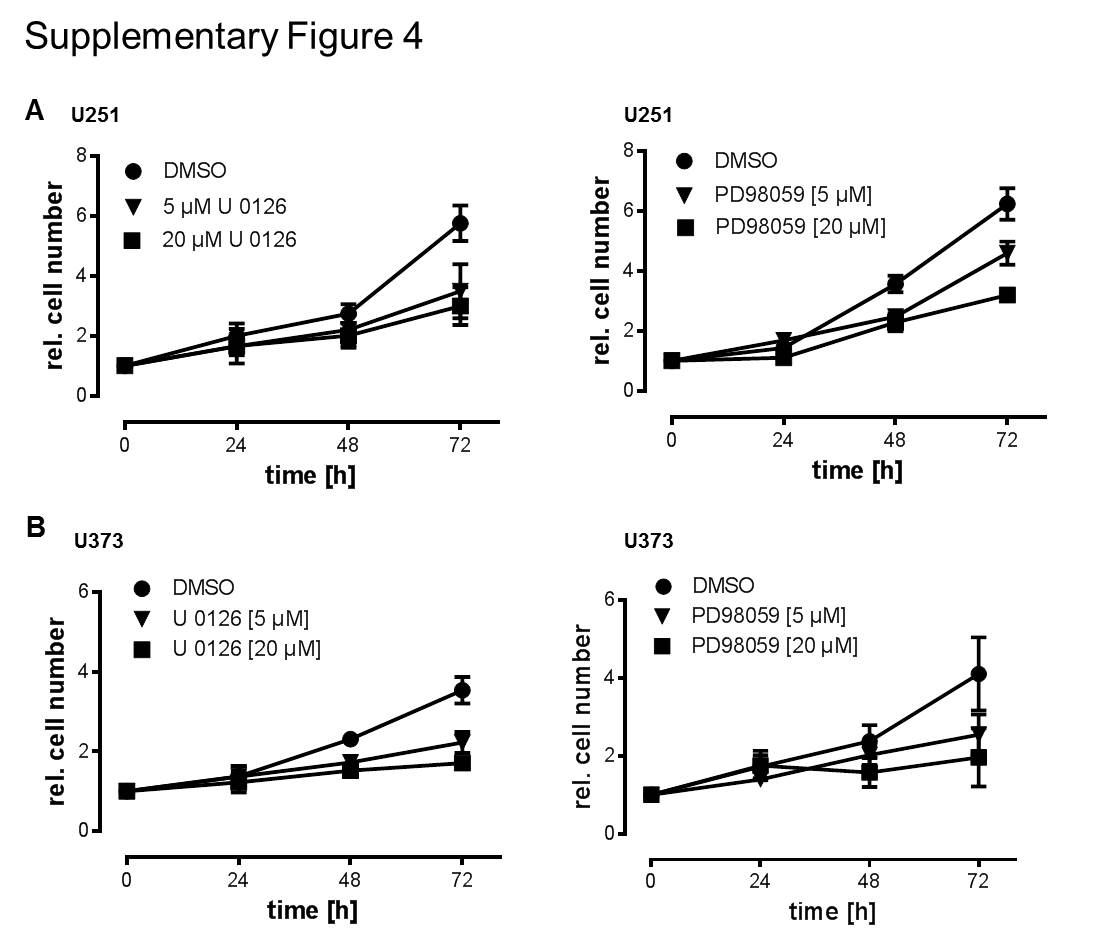

Supplement: Supplementary file 4 — Additional file 4: Figure S4. MEK inhibition blocks cell proliferation of U251 and U373 cells. (a) U251 cells and (b) U373 cells were treated with vehicle (DMSO), U0126 or PD98059 as indicated (n=3). Cells were trypsinized at 24, 48 and 72 hours post treatment and counted. Cell number at time point 0 hours was set to 1. [file 12935_2018_565_MOESM4_ESM.jpg]
